# Using Simpson’s diversity index to examine multidimensional models of diversity in health professions education

**DOI:** 10.5116/ijme.565e.1112

**Published:** 2016-01-03

**Authors:** Jacqueline E. McLaughlin, Gerald W. McLaughlin, Josetta S. McLaughlin, Carla Y. White

**Affiliations:** 1 University of North Carolina Eshelman School of Pharmacy, UNC Chapel Hill, North Carolina, USA; 2 Enrollment Management and Marketing, DePaul University in Chicago, Illinois, USA; 3 Walter E. Heller College of Business, Roosevelt University in Chicago, Illinois, USA; 4 Office of Innovative Leadership and Diversity, University of North Carolina Eshelman School of Pharmacy, UNC Chapel Hill, North Carolina, USA

**Keywords:** Diversity, measurement, decision support, institutional research, Simpson’s Diversity Index

## Abstract

**Objectives:**

This study explored new models of diversity for health professions education that incorporate multiple attributes and examined differences in diversity based on urbanicity, geographic region, and institutional structure.

**Methods:**

Simpson’s Diversity Index was used to develop race, gender, and interprofessional diversity indices for health professions schools in the United States (N = 318). Sullivan’s extension was used to develop a composite diversity index that incorporated multiple individual attributes for each school. Pearson’s r was used to investigate correlations between continuous variables. ANOVA and independent t-tests were used to compare groups based on urbanicity, geographic region, and Basic Carnegie Classification.

**Results:**

Mean (SD) for race, gender, and interprofessional  diversity indices were 0.36(0.17), 0.45(0.07), and 0.22(0.27) respectively. All correlations between the three indices were weak. The composite diversity index for this sample was 0.34(0.13). Significant differences in diversity were found between institutions based on urbanicity, Basic Carnegie Classification, and geographic region.

**Conclusions:**

Multidimensional models provide support for expanding measures of diversity to include multiple characteristics and attributes. The approach demonstrated in this study enables institutions to complement and extend traditional measures of diversity as a means of providing evidence for decision-making and progress towards institutional initiatives.

## Introduction

Creating diverse educational environments has important implications for institutions tasked with preparing students for 21^st^ century health care. ^1^ ^-^ ^3^ Research suggests that institutional culture associated with characteristics like race and gender can significantly impact the educational experiences of students and the training of aspiring health professionals. ^4^ Student attitudes and perspectives about health and culture, for example, are influenced by diversity-related interactions and experiences during school. ^5^ In addition, the growing emphasis on interprofessional health care teams and increasing complexity of multidisciplinary workplaces highlight the importance of interprofessional diversity (i.e. nursing, pharmacy, social work, medicine) in health professions education. ^6^ ^,^ ^7^ In one survey of medical, nursing, and pharmacy students, the majority reported positive attitudes towards interprofessional learning, citing development of team working skills, enhanced professional working relationships, and improved patient care as perceived benefits. ^7^

While the benefits of diverse educational environments are apparent, the conceptualization and measurement of diversity remains a challenge.  In a recent commentary, Chief Diversity Officer for the Association of American Medical Colleges Marc Nivet noted that, “Medical schools and teaching hospitals are shifting their strategies to better capture, leverage, and respond to the rich diversity of human talents and aptitudes” and that “initiatives are under way to integrate personal experiences and attributes into the existing metrics used to evaluate medical school applicants.” ^2^ As schools strive for a broadened conceptualization of diversity that reflects multiple attributes and characteristics, consideration must be given to how diversity is measured within the context of today’s changing education and health care systems. It is incumbent upon institutions to identify a way to broaden the measurement of diversity as a means of a) managing strategies aimed at achieving diversity and inclusion, b) identifying progress toward meeting diversity goals, and c) meeting legal requirements for targeted strategies with valid measures. ^8^ The challenge remaining is how to quantitatively incorporate multiple attributes (e.g., race, gender, and profession)  into a single metric that appropriately measures levels of multidimensional diversity within individual institutions.

Currently, the most common approach to measuring diversity in education is to count the magnitude or percentage of various demographic categories (e.g., race, ethnicity, gender, age) for a given population. This approach permeates education due to its simplicity and the lack of a viable alternative. However, these metrics cannot adequately describe attributes with multiple categories nor can they support simultaneous consideration of multiple individual attributes, such as intellectual attributes, geographic origin, language, and status (e.g., socio-economic, first-generation student, veteran and/or military). 

Using multidimensional models based on Simpson’s Index of Diversity and Sullivan’s extension can overcome the limitations of traditional diversity metrics based on magnitude or proportion. Simpson’s Index of Diversity, originally developed in the field of biology, models the probability that two randomly selected individuals will be from the same category based on the equation: 

(1) $$D=1-\sum_{}pi^{2}$$

over all i , where pi represents the proportion of each category i . ^9^ This index is capable of providing a single value for an attribute that is defined by two or more categories and addresses the fact that larger proportions of a category may, in fact, reduce diversity. In addition, when more than two categories are used to describe a single attribute, Simpson’s index can reflect the dispersion in multiple categories. This alleviates the need to identify a single category to reflect diversity. In the following example, Simpson’s index accounts for multiple professional degree programs at a single institution, with the composition represented as 20% nursing students, 10% pharmacy students, 65% medical students, and 5% other. Simpson’s Index provides a single value for describing the institution’s interprofessional diversity:

D = 1 - ((proportion of nursing students)^2^ + (proportion of pharmacy students)^2^ + (proportion of medical students)^2^ + (proportion of other)^2^) = 1 - (0.20 ^2^ + 0.10 ^2^ + 0.65 ^2^ + 0.05^2^) = 0.525

In this example, 0.525 represents the probability that two randomly selected individuals will be from the same professional degree program.  In 1973, Sullivan derived a composite index from Simpson’s Index of Diversity that combined six social and economic variables (Education, Income, Occupation, Housing, Ethnic, and Religious Orientation) using the following equation: ^10^ ^,^ ^11^

(2) $$A_{w}=1-\sum_{k=1}^{p}\left[\frac{{\left(Y_{k}\right)}^{2}}{V}\right]$$

Where, there are V variables, p categories and $Y_{k}$ proportions in each category.

When more than one attribute is used to measure diversity, Sullivan’s model represents the proportion of attributes on which a pair of individuals drawn at random will differ. Applying Sullivan’s model alleviates the need to identify a single attribute to represent diversity for multicultural and multi-disciplinary institutions that embody a range of backgrounds and perspectives in its mission statement and among its personnel.

The purpose of this study is to describe and demonstrate the adoption of a multidimensional diversity model that can enable health professions schools to address these challenges while monitoring progress toward key diversity initiatives. This demonstration is a first step toward a comprehensive measure of diversity that can accommodate attributes that are represented by more than two categories and concepts of diversity that incorporate multiple attributes (e.g., race, gender, and profession). In addition, the diversity metrics described here are used to examine differences in diversity based on an institution’s urbanicity, geographic region, and institutional structure. This analysis demonstrates the merits of considering institutional characteristics when conceptualizing student body diversity. 

## Methods

All data used in this study were collected from the Integrated Postsecondary Educational Data System (IPEDS). IPEDS is a publically-available federal database maintained by the United States (US) National Center for Educational Statistics (NCES) that includes data from all US institutions wishing to qualify students for federal grants. An institution was included in the study if it reported awarding 20 or more professional doctoral degrees in any health professions discipline in the 2011-2012 academic year. The variables used in this study were selected as examples of possible measures that an institution might use to describe its composition or to assess whether its composition is consistent with its mission or peers. For each institution in the study, the following variables were downloaded from the IPEDS website: number of professional doctoral degrees conferred in Chiropractic, Dental, Medical, Optometry, Osteopathic, Pharmacy, Podiatry, and Other; number of professional doctoral degrees conferred to Asian students, Black or African American students, Hispanic students, White students, and Other; number of professional doctoral degrees conferred to female students and to male students; level of urbanicity (i.e. population density of the area in which the school is located) categorized as urban, suburban, or town/rural; geographic region (i.e. physical location of the school within the US) categorized as East/Northeast, Great Lakes/Plains, Southeast, or West/Southwest; and Basic Carnegie Classification (i.e. whether or not the health professions school is affiliated with an undergraduate college or university) categorized as Special Focus Institutions – Medical schools and medical centers/other health professions schools or all other institutions. Urbanicity, geographic region, and Basic Carnegie Classification were selected because they are considered fundamental to an institution’s mission and are commonly used in peer comparisons of US schools. ^12^ The total sample size for this study was 55,290 students from 318 institutions.

For each institution in this study, a race index, gender index, and interprofessional diversity index was computed using the formula for Simpson’s Diversity Index. A race index, for example was calculated according to the following formula:

D = 1 - ((proportion of Asian students)^2^ + (proportion of Black students)^2^ + (proportion of Hispanic students)^2^ + (proportion of White students)^2^ + (proportion of other)^2^)

A composite diversity index (CDI) was calculated for each institution per Sullivan’s model using proportions from race, gender, and profession. In this study, the CDI represents the proportion of the three attributes (race, gender, and profession) on which a pair of individuals drawn at random from a single institution will differ.

All quantitative data analysis was conducted in SPSS for Windows, Version 21 (IBM, 2013). Continuous data is represented as mean, standard deviation (SD). Pearson’s r was used to investigate correlations between continuous variables. ANOVA and independent t-tests were used to compare groups based on urbanicity, geographic region, and Basic Carnegie Classification. Statistical significance was established at α=0.05.

## Results

The demographic characteristics and corresponding diversity indices for the 318 health professions school included in this study can be seen in Table 1 . These institutions awarded a total of 55,290 health professions degrees in 2012 (mean 177.21(154.93) degrees per institution) and 144 institutions awarded health professions doctoral degrees in more than one health professions profession. The mean (SD) race, gender, and profession diversity indices for all institutions were 0.36(0.17), 0.45(0.07), and 0.22(0.27) respectively.

**Table 1:** Characteristics of students in US institutions (N=318)

Characteristic		Mean (SD)
Race		
	Asian (%)	14.82 (14.33)
	Black(%)	6.18 (11.02)
	Hispanic(%)	4.51 (5.93)
	White(%)	71.53 (20.53)
Race Diversity		0.36 (0.17)
Gender		
	Female (%)	58.87 (12.06)
Gender Diversity		0.45 (0.07)
Interprofessional*		
	Chiropractic (%)	4.40 (20.14)
	Dental (%)	4.87 (12.87)
	Medical (%)	24.24 (34.78)
	Optometry (%)	3.54 (17.64)
	Osteopathic (%)	5.31 (20.03)
	Pharmacy (%)	20.20 (33.51)
	Podiatry (%)	1.29 (10.06)
	Other (%)	36.15 (41.63)
Discipline Diversity		0.22 (0.27)
Composite Diversity Index		0.34 (0.13)

*Mean (SD) for each discipline represents the average percentage of discipline-specific doctorates awarded per health professional doctorate awarded per institution.

All correlations between the three indices were less than 0.3 and considered weak relationships. The CDI for this sample was 0.34(0.13). Significant differences in race, gender, and profession diversity and the CDI were found based on institutional characteristics. As seen in Table 2 , institutions located in town/rural areas tended to have lower scores associated with race diversity (p = 0.005), profession diversity (p = 0.015), and CDI (p = 0.002).  When compared to institutions associated with an undergraduate college or university, schools with a Basic Carnegie Classification of Special Focus Institution (medical schools or other health professions schools not affiliated with a college or university) had higher race diversity (p < 0.001), gender diversity (p < 0.001), and CDI (p = 0.002).  Further, race diversity (p<0.001) and CDI (p < 0.006) differed significantly by geographic region, with schools in the West/Southwest demonstrating higher race diversity and CDI scores and schools in the Great Lakes/Plains reporting lower race diversity and CDI scores.

**Table 2:** Diversity indices* for US health professions institutions (N = 318)

Variable		Race Diversity Mean (SD)	*p-value*	Gender Diversity Mean (SD)	*p-value*	Profession Diversity Mean (SD)	*p-value*	CDI Mean (SD)	*p-value*
Urbanicity**									
	Urban (n = 217)	0.37(0.17)	0.005	0.46(0.07)	0.974	0.25(0.28)	0.015	0.34(0.13)	0.002
	Suburban (n = 62)	0.37(0.17)		0.45(0.07)		0.20(0.27)		0.34(0.13)	
	Town/Rural (n = 35)	0.27(0.18)		0.45(0.06)		0.11(0.21)		0.28(0.11)	
Geographic Region**									
	East/Northeast (n = 83)	0.39(0.18)	<0.001	0.45(0.07)	0.624	0.19(0.25)	0.589	0.35(0.13)	0.006
	Great Lakes/Plains (n = 85)	0.28(0.15)		0.45(0.08)		0.22(0.28)		0.32(0.13)	
	Southeast (n = 81)	0.34(0.15)		0.46(0.07)		0.21(0.27)		0.33(0.11)	
	West/Southwest (n = 69)	0.45(0.16)		0.46(0.06)		0.25(0.28)		0.39(0.13)	
Carnegie Category†									
	Special Focus (n = 80)	0.43(0.14)	<0.001	0.48(0.04)	<0.001	0.15(0.23)	0.216	0.38(0.12)	0.002
	All Others (n = 238)	0.34(0.18)		0.45(0.07)		0.20(0.27)		0.33(0.13)	

*Diversity indices calculated using Simpson’s Index of Diversity. **One-way ANOVA with Bonferonni adjustment used to compare groups. †Independent t-test used to compare groups.

## Discussion

Measurement of diversity is a topic of increasing importance to health professions education. Educators have been called upon, for example, to “take an explicitly wider view of the influence of what has sometimes been called the hidden curriculum.” ^4^

While interest and attention has increased, the use of magnitude and proportion to measure diversity continues to prevail. The approach described in this paper enables institutions to overcome the limitations associated with monotonic measures by a) capturing the dispersion of an attribute with multiple categories and b) accommodating multiple attributes with a single metric. By implementing a multidimensional diversity model, institutions can expand traditional compositional metrics, evaluate the efficacy of diversity initiatives, and monitor longitudinal changes in institutional diversity in an effort to enhance the experiences of students, faculty, staff, and patients.

In this study, variable selection was limited to those attributes collected and reported by the US federal government within IPEDS. As institutions consider this approach, it should be noted that actual attributes of interest will most likely vary from institution to institution as influenced by the mission, vision, and goals of the institution. Institutions should be mindful of other data sources, both internal and external, that can contribute to their own measurement and interpretation of diversity. Each institution must give careful consideration to how an attribute will be measured, which attributes should be included, and how the model results should be interpreted within the context of the institution. Health profession institutions might also consider building internal databases that include characteristics not otherwise available.

In addition, the findings of this study suggest that institutional characteristics such as geographic region and Basic Carnegie Category are important factors in understanding diversity in health professions education. Differences found based on institutional characteristics provide guidance for institutions to benchmark their diversity against similar or aspirational peers. Specifically, results from this study suggest that institutions should consider urbanicity, geographic region, and Basic Carnegie Classification when selecting peers for benchmarking various types of diversity. The findings from this study support other diversity research in higher education that identifies differences based on location and classification. ^13^

While the indices described and examined in this paper provide a mechanism for measuring multidimensional diversity, the authors recognize that traditional measures of compositional diversity still play an important role in understanding and monitoring diversity in health professions education. Monitoring the magnitude and proportion of underrepresented groups, for example, should remain a priority for our professions. Multidimensional metrics should not be used at the exclusion of relevant proportion data; rather, these indices should complement and extend traditional measures as a means of providing evidence for decision-making and progress towards institutional initiatives.

Diversity indices, including a composite multidimensional metric like the CDI, can support strategic efforts to enhance diversity and inclusion. It is our hope that the approach described in this paper enables health professions schools to establish core educational diversity goals, measure progress towards meeting those goals, and implement processes that move rhetoric about multidimensional diversity from the mission to operational reality. As Nivet notes, “medical schools and teaching hospitals must acknowledge diversity as a strategic imperative.” ^2^

## Conclusions

As our educational and health care systems become increasingly complex, it is not surprising that interest in diversity continues to grow. The findings from this study challenge us to expand our description of diversity to include multiple individual and institutional characteristics. While attributes like race, gender, and profession clearly warrant consideration, institutions should also consider including those attributes that speak to their own core diversity goals and institutional mission. The approach described here provides a mechanism for aligning diversity measurement with institutional initiatives with the hopes of enhancing the experience of students, faculty, staff, and patients and fostering the innovation needed within our rapidly evolving healthcare systems. 

### Conflict of Interest

The authors declare that they have no conflict of interest.
